# Seasonal Shifts in Diet and Gut Microbiota of the American Bison (*Bison bison*)

**DOI:** 10.1371/journal.pone.0142409

**Published:** 2015-11-12

**Authors:** Gaddy T. Bergmann, Joseph M. Craine, Michael S. Robeson, Noah Fierer

**Affiliations:** 1 Department of Ecology and Evolutionary Biology, University of Colorado, Boulder, Boulder, Colorado, United States of America; 2 Cooperative Institute for Research in Environmental Science, University of Colorado, Boulder, Boulder, Colorado, United States of America; 3 Division of Biology, Kansas State University, Manhattan, Kansas, United States of America; 4 Fish and Wildlife Conservation Biology, Colorado State University, Fort Collins, Colorado, United States of America; 5 USDA APHIS, National Wildlife Research Center, Fort Collins, Colorado, United States of America; Smithsonian Conservation Biology Institute, UNITED STATES

## Abstract

North American bison (*Bison bison*) are becoming increasingly important to both grassland management and commercial ranching. However, a lack of quantitative data on their diet constrains conservation efforts and the ability to predict bison effects on grasslands. In particular, we know little about the seasonality of the bison diet, the degree to which bison supplement their diet with eudicots, and how changes in diet influence gut microbial communities, all of which play important roles in ungulate performance. To address these knowledge gaps, we quantified seasonal patterns in bison diet and gut microbial community composition for a bison herd in Kansas using DNA sequencing-based analyses of both chloroplast and microbial DNA contained in fecal matter. Across the 11 sampling dates that spanned 166 days, we found that diet shifted continuously over the growing season, allowing bison to take advantage of the seasonal availability of high-protein plant species. Bison consumed more woody shrubs in spring and fall than in summer, when forb and grass intake predominated. In examining gut microbiota, the bacterial phylum *Tenericutes* shifted significantly in relative abundance over the growing season. This work suggests that North American bison can continuously adjust their diet with a high reliance on non-grasses throughout the year. In addition, we find evidence for seasonal patterns in gut community composition that are likely driven by the observed dietary changes.

## Introduction

North American bison (*Bison bison*) are a keystone species in the grasslands of the Great Plains, where their feeding, migration, and wallowing activities promote plant diversity [1]. Bison are considered primarily grazers, meaning they consume mostly graminoids (grasses and sedges) [2–5]. The bison diet varies across different geographic regions, depending on the type and abundance of potential forage available. For example, in northern habitats, bison diets are dominated by cool-season graminoids [3]. Yet bison may also browse, feeding on shrubs such as *Salix* as their primary forage when graminoids are scarce [6,7].

In general, herbivores have been shown to prefer fresh shoots, because they are often lower in plant secondary compounds and richer in protein, making such plants more palatable, easier to digest, and more nourishing [8–10]. Phenologically driven pursuit of new growth was first observed in geese [11–14], and then in deer [8,15–17]. Bison prefer newly produced shoots as well, including those on mowed areas [18], burned areas [19–22], and prairie dog towns [23–25]. Despite their historic keystone role in North American grasslands, little is known about how the bison diet changes seasonally. Bison have been observed exhibiting seasonal variation in diet, continuing to feed on graminoids [3,26], but also incorporating forbs, woody plants, and even lichen into their diet [27]. These temporal shifts in the bison diet are likely due to the changing nutritional quality of plants, and likely important to understanding the health of bison, as well as the impacts of bison on grassland ecosystems.

Along with our poor understanding of the seasonal patterns of bison diet, we do not know if bison gut microbial communities shift in response to changes in diet. Like all ruminants, bison rely on symbiotic microbes to help them digest vegetation [28]. Digestion begins in the reticulorumen, and continues in other parts of the digestive tract, concluding in the colon [5]. Domestic cattle (*Bos taurus*) on pasture or forage retain a diverse, benign gut microbiota associated with a healthy digestive tract. The few studies on the seasonal patterns of gut microbiota in wild herbivores have found changes in gut community composition that are associated with changes in diet [29,30]. If changes in gut microbial communities are associated with dietary shifts in bison, this would suggest that microbes respond to changes in nutritional quality, antinutritional secondary metabolites, or plant species composition of the diet [31–33].

Understanding seasonal variation in plant consumption will increase our understanding of bison’s dietary needs. Documenting concurrent shifts in gut microbiota will help us establish baseline rates of change in gut community composition throughout the year, and assess whether microbial shifts are associated with dietary changes. In order to better understand seasonal patterns of diet and gut microbial community composition, bison fecal material was collected from adult and subadult bison in a Kansas prairie approximately every 14 d during the growing season. An herbivore’s diet is affected by both plant availability and forager preference for different plant species [34–36], so both plant availability and the preferences of bison will affect the amounts of different plants ultimately consumed by bison. However, computing preference indices requires assessing plant abundance and dispersion in the environment [37,38]. Diet and gut microbial community changes over time were reconstructed using DNA sequencing-based analyses of both plant chloroplast DNA and the 16S rRNA gene of bacteria and archaea. These data were used to address two main questions: to what degree does the bison diet change throughout the growing season, and do temporal changes in diet drive corresponding shifts in gut microbial community composition?

## Materials and Methods

### Study design

The Konza Prairie Biological Station is a private 3,487-ha native tallgrass prairie preserve in the Flint Hills of northeastern Kansas (39.08 N, 96.57 W). American bison (*Bison bison*) were reintroduced to the preserve in 1987, and now about 300 of them live in a fenced range of about 970 ha. To study their diet and gut microbiota, fecal samples were collected every other week from adult and subadult bison (three male and three female) during the period of April 17–September 30, 2011. Samples were collected from the first individuals observed to defecate during each collection trip, which may or may not have been the same individuals from one trip to the next. Fecal samples were sampled 48 times and divided into two demographic groups: 24 adult or subadult males, and 24 adult or subadult females. Collectors waited at a distance for the bison to defecate, and once they moved away, fresh fecal material was sampled with an inverted plastic bag, taking care to avoid contamination from soil underneath. Fecal samples were transported on ice, and stored at -20°C. Because only bison fecal material and vegetation were collected for the present study, no Institutional Animal Care and Use Committee (IACUC) approval was required. John Briggs, Director of Konza Prairie Biological Station, was the authority who reviewed our procedures and granted us permission to collect samples.

### Diet analyses

We used analyses of plant chloroplast DNA in feces to infer which plant species the bison had consumed, and to track changes in these plants’ relative abundance in the diet over time. This method, known as the *trn*L approach [39], uses the P6 loop of the *trn*L (UAA) intron to assay diet in herbivores [40]. We used the *trn*L g-h primer pair, whose targeted gene region is only 10–146 bp long, making it likely that the gene region can be amplified and sequenced from feces after passage of the plant material through the bison gut. The amplified region is also highly variable, so it can be used to differentiate many plant species [41]. An identical approach has been used previously for non-invasive analysis of herbivore diet [42–44].

To match the *trn*L sequences to plant species, we created a *trn*L reference library for the site by acquiring representative samples of 73 plant species to which the bison had access. Single specimens of plants were collected in the field and placed in sealable plastic bags. They were identified and stored in the freezer at -20°C. To extract DNA from plants, vegetation samples were finely chopped with a sterile razor prior to DNA extraction. To extract DNA from bison feces, sterile swabs were dipped in thawed fecal samples before also being placed in reaction wells. For both sample types, DNA was extracted using the MO BIO PowerSoil®-htp 96 Well Soil DNA Isolation Kit [45].

To amplify the *trn*L fragment from the plants and fecal samples for barcoded pyrosequencing, we followed [46], except with the primer pair g-forward/h-reverse, which targets the *trn*L fragment [41]. The forward primers included the Roche 454-B pyrosequencing adapter, while the reverse primers included the Roche 454-A sequencing adapter and a 12-bp barcode that was unique to each sample. Amplicons from the triplicate reactions were combined, cleaned, quantified, and pooled in equimolar concentrations. The pooled sample was sent to the University of South Carolina for sequencing on a Roche 454 automated sequencer.

Sequence data were processed using the QIIME pipeline [47]. Sequences were assigned to specific plant samples based on their unique barcodes, and sequences were clustered at 100% similarity for each plant species. A reference library of *trn*L sequences was constructed using consensus sequences from identified plant specimens. For plants with at least 10 representative sequences, the consensus sequence was the one that made up at least 40% of sequences in a given sample, and was at least 30% more abundant than the second-most common sequence. As some of the plant species shared identical *trn*L sequences, our collection of 73 plant species was represented by 44 *trn*L sequences, with 32 species (44%) having unique representative sequences (S1 Table). For the remaining 41 plants (56%), two or more species shared sequences, meaning they could not be distinguished if detected [39]. The *trn*L sequences from fecal samples were matched against this *trn*L reference database using the BLAST algorithm at ≥ 98% similarity over the entire length of the reference sequence [48].

Because different fecal samples have different *trn*L sequence counts, we rarefied down to 50 sequences, to compare all samples at an equivalent sequencing depth. Chloroplast *trn*L sequences in feces might be affected not only by the abundance of plants from which they came, but also by chloroplast and gene copy number in living plants, and by the digestibility of plants in the herbivore digestive tract. Thus, the sequence of *trn*L genes in feces depends not only on the plant species consumed, but also on the density of chloroplasts in the plant biomass, and the degree to which chloroplasts of different species are digested. One feeding trial demonstrated no consistent bias between the percentage intake of biomass and percentage of sequences in fecal matter of sheep [49]. Given that *trn*L is a chloroplast gene with no known variation in copy number in the chloroplast genome, we assume for the purposes of the present study that the percent of sequences recovered from fecal matter is proportional to the relative intake of chloroplasts from the different plant taxa. Thus, although the proportions of different plants in the diet may not correspond perfectly to proportions of different *trn*L sequences in feces, the *trn*L approach allows us to document relative changes in the composition of specific plant taxa over time [49].

### Microbial analyses

We also used the collected fecal samples to track shifts in gut microbiota across the sampling period, and to assess if the changes in gut microbial communities are associated with temporal shifts in diet. We sequenced a portion of the 16S rRNA gene from bison fecal DNA to study bacterial and archaeal community composition in the colon. Fecal DNA was extracted as described above. Amplification and sequencing followed the approach used previously [46]. Briefly, we used the 515F/806R primer pair containing Illumina adapters, with a 12-bp error-correcting barcode unique to each sample on the reverse primer. The V4–V5 region of the 16S rRNA gene amplified by this primer set is well-suited to accurate phylogenetic placement of bacterial and archaeal sequences [50]. Together, these primers are expected to amplify nearly all bacterial and archaeal taxa with few biases [51].

After quantification and pooling, the amplicons were sequenced on an Illumina MiSeq instrument at the University of Colorado Genomics Core Facility with the 2 × 100 bp paired-end protocol [52]. We used the QIIME pipeline for data analysis on the forward reads only [53]. Quality filtering and processing of reads was performed following [52]. Bacterial 16S rRNA sequences were clustered at 97% similarity, and a representative sequence from each OTU was classified against the RDPII database [54]. Due to unequal numbers of 16S rRNA sequences in each fecal sample, we rarefied down to a depth of 15,000 sequences to compare all samples at an equivalent sequencing depth.

### Statistical analysis

To test the effect of time on diet, plants were grouped into three functional groups, corresponding to the following growth habits: graminoids (grasses and sedges), forbs (non-graminoid herbaceous plants), and woody vegetation (shrubs and trees). A small number of *trn*L sequences (≤ 2%) could not be distinguished between forbs and graminoids, and were not included in downstream analysis. OTU tables for both *trn*L data and the 16S rRNA data were square root-transformed, and the Bray-Curtis method was used to generate distance matrices for multivariate statistical analysis, namely principal coordinates analysis (PCoA) and PERMANOVA in the PRIMER 6.1.12 & PERMANOVA + 1.0.2 software package [55]. PERMANOVA indicated that diet and gut community composition were not significantly different between the sexes or between adults and subadults (P = 0.53), so we combined all these non-calf bison prior to further analysis. We used repeated measures ANOVA with Bonferroni correction to test for the effect of time on the relative abundance of the three plant functional groups, the ten most abundant plant species, and the three most abundant microbial phyla using the Tukey’s Honest Significant Difference (HSD) post-hoc test [56,57]. We used linear regression to test the relationship between the proportions of plant functional groups and microbial phyla. Finally, we fit quadratic curves to the mean proportions of each plant growth habit over the growing season in the bison diet. Repeated measures ANOVA, Tukey’s HSD, linear regression, and quadratic fitting were all conducted in the R statistical package [58].

## Results

### Diet

**Fig 1:**
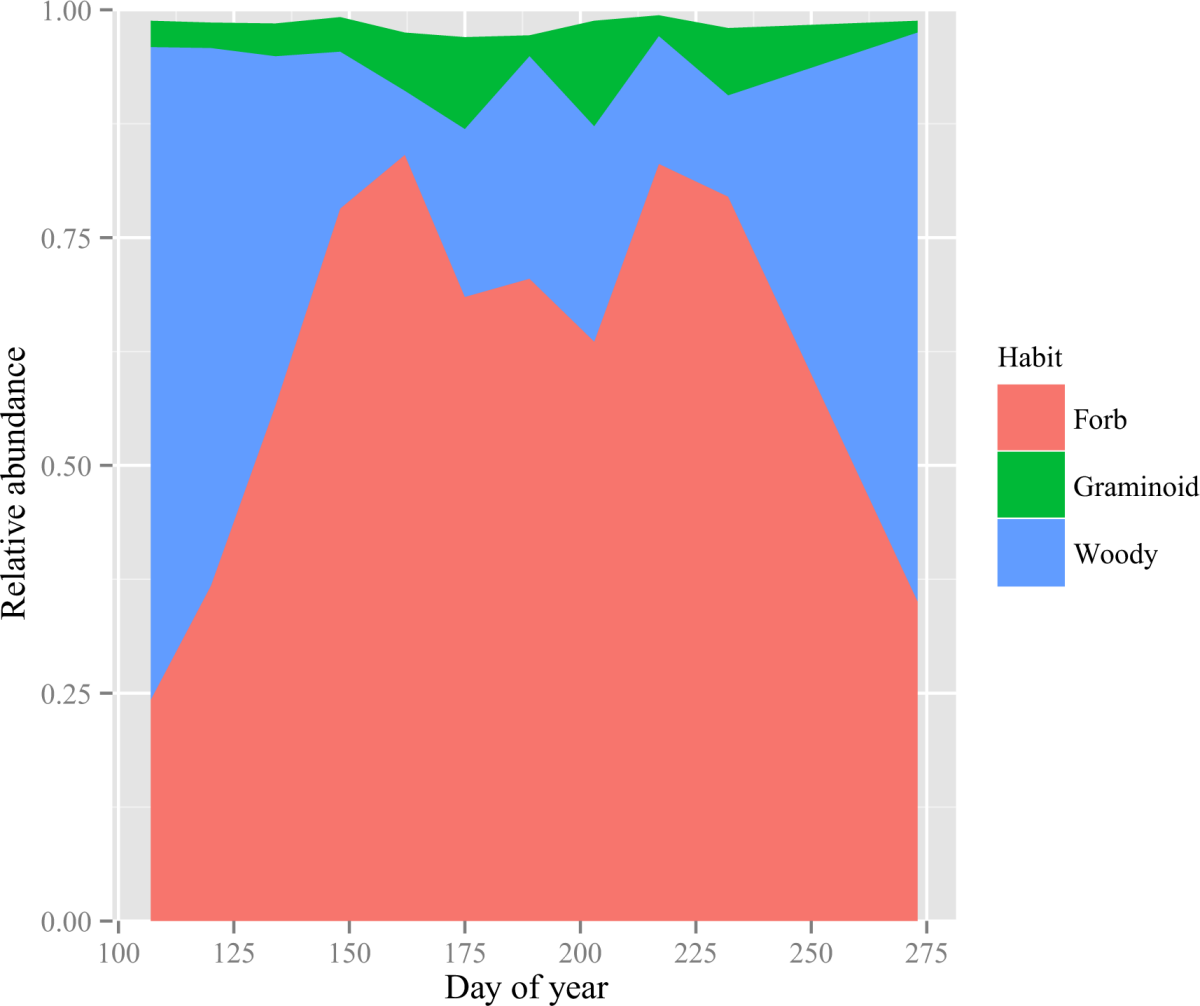
Changes in plant diet as indicated by shifts in the relative abundance of *trn*L chloroplast gene sequences. Plant species were grouped by growth habit (forb, graminoid, and woody). The effect of time in each dataset was tested using repeated measures ANOVA with Bonferroni correction. All three functional groups exhibited significant temporal change in proportion of the diet (*P* < 0.05).

Across 11 sampling dates spanning 166 days, analysis of bison feces revealed 44 unique *trn*L sequences, representing up to 73 different plant species in the bison diet. Dietary composition varied seasonally (*P* < 0.001, S2 Table and S1 Fig). The four most abundant clusters (groups of species with a single *trn*L sequence) were the *Achillea* forb cluster (*Achillea millefolium*, *Chloris verticillata*), the *Ageratina* forb cluster (*Ageratina altissima*, *Ambrosia artemisiifolia*, *Ambrosia psilostachya*, *Ambrosia trifida*, *Antennaria neglecta*, *Echinacea angustifolia*, *Helianthus maximiliani*, *Helianthus tuberosus*, *Ratibida pinnata*, *Silphium laciniatum*, *Taraxacum officinale*, and *Euphorbia corollata*), the *Andropogon* graminoid cluster (*Andropogon gerardii*, *Bothriochloa bladhii*, *Bothriochloa laguroides*, and *Schizachyrium scoparium*), and the *Oligoneuron* forb cluster (*Oligoneuron rigidum*, *Solidago canadensis*, *Solidago missouriensis*, *Solidago speciosa*, *Symphyotrichum ericoides*, and *Symphyotrichum laeve*). Plotting the relative abundance of plants by growth habit over time (Fig 1), bison consumption of woody vegetation was greater early and late in the growing season than in mid-season (*P* < 0.001, quadratic fit: y = 0.55–0.0025*DOY + 7.31*10^−5^*(DOY-178.2)^2^; DOY = Day of Year). *Ceanothus herbaceus*, an actinorhizal N_2_-fixing shrub, was the primary woody species consumed. This species exhibited a significant change in proportion over the growing season (*P* < 0.001), contributing on average as many as 56% of the sequences in the spring and 60% in the fall.

**Fig 2:**
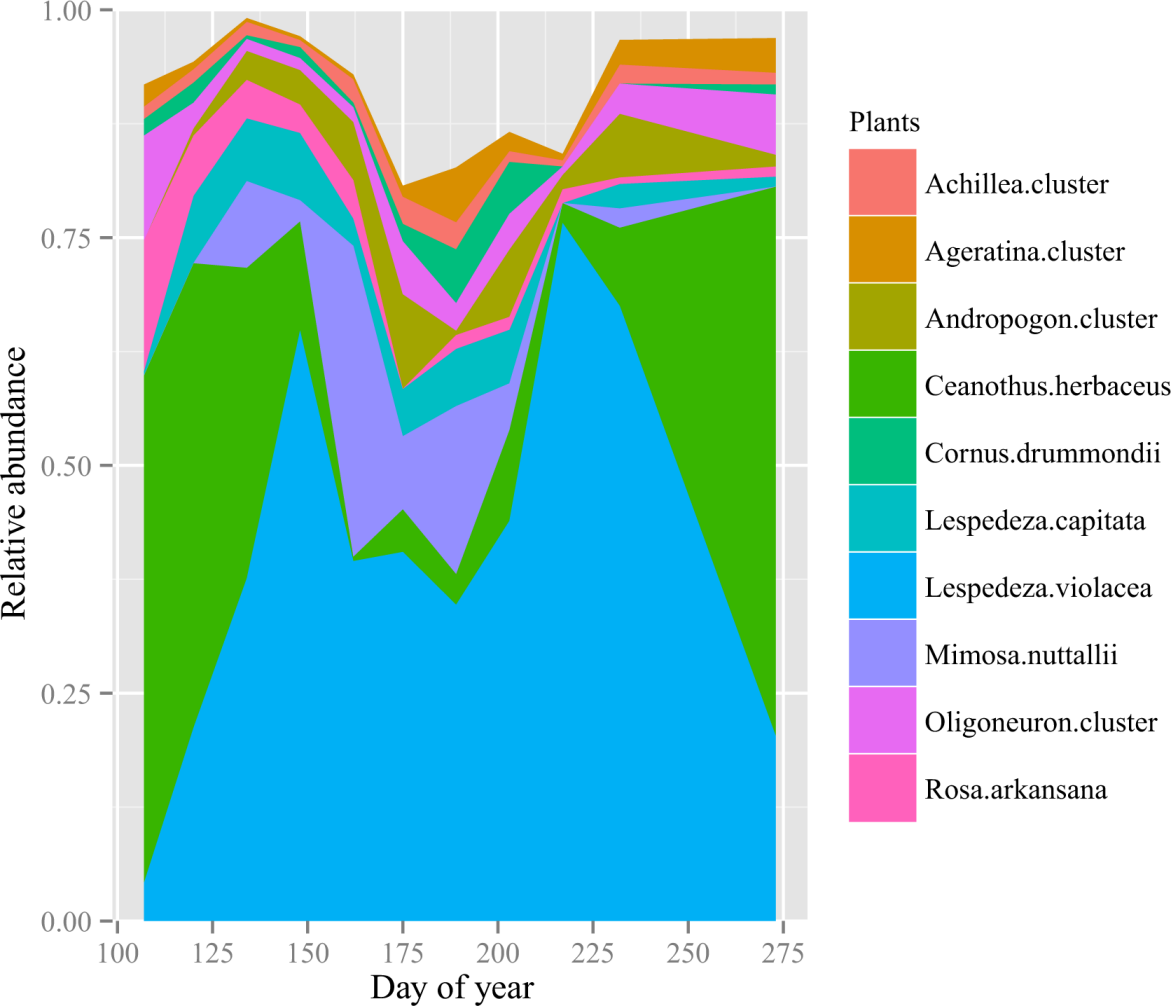
Plant relative abundance as indicated by sequencing of *trn*L chloroplast genes. Plants were identified to species by matching database sequences at the 100% level. See Results for the identity of species in clusters. The effect of time in each dataset was tested using repeated measures ANOVA with Bonferroni correction. Three taxa (*Lespedeza violacea*, *Ceanothus herbaceus*, and the *Oligoneuron* forb cluster) exhibited significant temporal change in proportion of the diet (P < 0.01).

Consumption of herbaceous species peaked in the middle of the season (Fig 1, y = 0.44 + 0.0024*DOY–7.3*10^−5^*(DOY-178.2)^2^ *; P <* 0.05). At their greatest relative abundance, 84% of the sequences on average could come from forbs. *Lespedeza violacea*, a legume, was the most abundant forb during the summer. Its proportion in the diet also changed significantly over time (*P* < 0.05), with as many as 77% of the dietary sequences on average coming from this species. The *Oligoneuron* cluster was another predominant group of forbs to exhibit significant temporal change (*P* < 0.05). The percentage of sequences derived from grasses was never higher than 12% on average, and grasses were consumed more in summer than in spring or fall (P < 0.001, Fig 2). The *Andropogon* cluster was the most abundant of the graminoid *trn*L clusters, but individually it did not show significant change over time (*P* > 0.1).

### Gut microbiota

**Fig 3:**
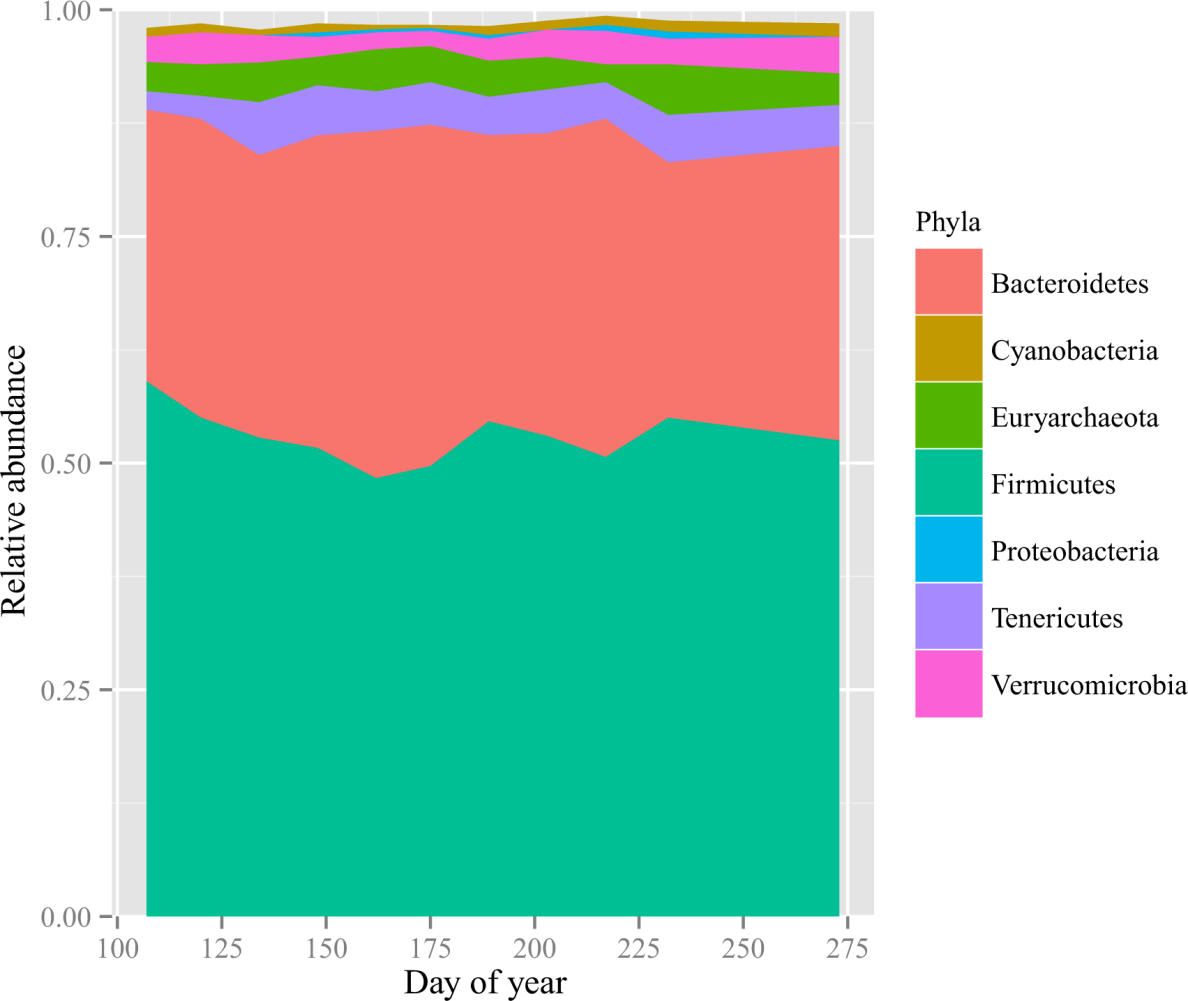
Relative abundance of microbial phyla as indicated by sequencing of 16S ribosomal RNA genes. The effect of time on each microbial phylum was tested using repeated measures ANOVA with Bonferroni correction. The phylum *Tenericutes* exhibited a significant shift in relative abundance over the growing season (*P* < 0.001).

Using 16S rRNA gene sequence data, we identified 46,061 bacterial species, 355 archaeal species, and 7 unclassified microbial species in the sampled bison during the growing season. Gut microbial community composition varied over time (P < 0.001, S2 Table and S1 Fig). The three most abundant microbial phyla were *Firmicutes* (53% of sequences on average), *Bacteroidetes* (33%) and *Tenericutes* (4%). The *Firmicutes* phylum was dominated by taxa within the *Clostridiales* order, while taxa within the *Bacteroidetes* order were the dominant *Bacteroidales*. Finally, *Tenericutes* consisted entirely of taxa within the *Mollicutes* class, which was comprised entirely of the putative order RF39 (S3 Table). Although there was no significant relationship between the proportion of sequences from graminoid, forb, or woody plants and the relative abundance of *Firmicutes*, *Bacteroidetes*, or *Tenericutes* (*P >* 0.05), the phylum *Tenericutes* exhibited significant temporal change in relative abundance over the growing season, increasing more than twofold from about 2% of the gut microbial community in April to about 5% in May (P < 0.001, S3 Table and Fig 3).

## Discussion

### Diet

Bison are considered to be primarily grazers [2–4], but in this study the proportion of grass chloroplast sequences recovered from fecal samples was relatively low during the summer. This could imply that the proportion of chloroplasts ingested from grass biomass was relatively low. The present study did not quantify forage availability or compute preference indices, but previous research has shown good correspondence between the relative amounts of biomass and sequence abundances in feeding trials [49]. However, the relatively low percentage of grass *trn*L sequences could be due to preferential degradation of grass DNA during passage through the gastrointestinal tract. Alternatively, differences in protein concentration among plants are likely to be associated with differences in chloroplast density, so high-protein plants could be over-represented in the *trn*L libraries relative to biomass intake. Further research is needed to determine if the proportion of *trn*L sequences in fecal material accurately reflects the consumption of the respective plants. Because some plant species could not be distinguished using the current method, future work could improve taxonomic resolution by using a different *trn*L primer pair than g-h, such as c-h or c-d, which produce longer sequences [41], or by incorporating additional loci, such as *rbc*L, into the identification system [59,60]. Nevertheless, we can use the data presented here to assess changes in the relative intake of plant taxa over time.

Our results from *trn*L sequencing indicate that plains bison at Konza shift their diet among high-protein plant species seasonally. In the spring and fall, intake of *Ceanothus herbaceus*, an N_2_-fixing shrub, is relatively high. During the summer, bison consumption of N_2_-fixing legumes like *Lespedeza violacea* and *Mimosa nuttallii* peaks (Fig 2). This pattern may be driven by plant phenology, as changes in the inferred diet of the bison roughly corresponded to plant phenology at this site [61]. Herbivores generally prefer to feed on new growth, because fresh shoots of a given type of plant are higher in moisture and nutrient content (including protein), and lower in fiber and secondary metabolites, making them both more palatable and more nutritious, even for grazers [62]. The bison’s diet was likely influenced by both the availability of and preference for nutritious vegetation [26,63], with the bison appearing to favor high-protein plant species and life stages [24,27,49].

Many ruminants in both the Afrotropic and Holarctic ecozones exhibit seasonal variation in plant consumption [64–67], and ruminant species that do not change diet with the seasons change location instead [17,68–70]. Domestic cattle [71], European wisent (*Bison bonasus*) [72], and North American bison [21,73] are all among the temperate ruminants that take advantage of preferred vegetation during the summer, and tolerate suboptimal vegetation during the winter. Our results appear to corroborate this pattern in bison. Fresh shoots of forbs and graminoids in summer are likely relatively high in protein and relatively low in secondary metabolites [62,74,75]. Woody shrubs selected in spring and fall, such as *Ceanothus*, are potentially high in secondary metabolites [74–76], but also high in protein [77–79]. Thus, like other ruminants [80–82], bison appear to exploit more nutritious forage during the growing season, but still accept less nutritious forage outside the growing season [27,63].

The relative abundance of eudicots in the bison diet raises questions about the degree to which bison should be considered obligate grazers [4,83,84]. Their broad mouth, massive shoulders, and low-slung head allow them to crop vegetation close to the ground [85–87], and their large reticulorumen facilitates digesting large amounts of low-nutrient graminoids [88]. However, our work shows that North American bison, like wisent and cattle, supplement their diet with more nutritious forbs and woody species throughout the growing season. *Bison* and *Bos* species, like their common ancestor *Leptobos*, have adaptations for grazing [89]. However, today’s plains bison subspecies (*B*. *bison bison*) is thought to have a more grass-dominated diet than fossil *Bison* populations [90,91], the contemporary wood bison subspecies (*B*. *bison athabascae*) [92], and European wisent (the closest living relative of the American bison species). Yet this does not mean that plains bison do not browse or utilize non-graminoids [40]. The molecular evidence presented here suggests that the dependence of *Bison* species on grasses might be more labile than previously thought.

### Microbiota

As in other mammals, *Firmicutes* and *Bacteroidetes* were the most abundant bacterial phyla in the bison digestive tract [93–95]. As in other studies showing that gut microbial community composition can be structured by diet [96–99], we found correspondence between major microbial phyla and dietary composition. In the change from spring to summer, *Tenericutes* became significantly more abundant (P < 0.05, S3 Table and Fig 3). This increase may be driven by members of this phylum that preferentially metabolize simple sugars [100], which could have been associated with higher caloric and protein yields in the bison diet during summer and fall [101]. Together, these results highlight that there are subtle but significant shifts in gut bacterial community composition that correspond to seasonal changes in the bison diet. The changes we observed are unlikely to be due to the influx of bacteria adhering to ingested plant matter, for although some microbes (and their DNA) can survive passage through the digestive tract [102], the vast majority of dominant bacterial taxa in fecal samples are rarely found in the phyllosphere (see [97] and [103]). Factors contributing to this difference include the harsh environment of the stomach [104], differences between the enteric and ambient environments [105], and strong competition from established members of the gut microbial community in mature animals [106–108]. Thus, the shift in microbial communities observed here are most likely to reflect shifts in enteric communities more than phyllosphere communities.

Although we do not know the impact of this microbial community shift on bison health, the results of the present study indicate that gut microbial communities are not static, and that even presumably healthy animals can experience significant temporal variability in gut microbial community composition.

## Supporting Information

S1 Fig Changes in the composition of (A) diet and (B) gut microbiota over time, as indicated by *trn*L chloroplast genes and 16S ribosomal RNA genes, respectively. Plot depicts primary axis of principal coordinate analysis (PCoA) for relative abundance data. In PCoA, the first axis accounts for the greatest amount of variation in the dataset. The effect of time in each dataset was tested using PERMANOVA (*P* < 0.001 in both cases).
(TIF)

S1 Table Summary of 44 operational taxonomic units based on plants sharing the same *trn*L sequences, representing 73 separate plant species. (XLSX)

S2 Table Table showing results from the principal coordinate analysis (PCoA). (XLSX)

S3 Table Heat map showing relative abundance of microbial phyla over time, based on sequences recovered from the feces of free-ranging bison. Numbers indicate mean relative abundance. Colors indicate z-score, with red representing positive z-scores, blue representing negative z-scores, and brightness of color representing absolute value of z-scores.
(TIF)
