# Supplementary figures and images for: Seasonal Shifts in Diet and Gut Microbiota of the American Bison (Bison bison)
Source: PLoS One. 2015 Nov 12;10(11):e0142409. doi: 10.1371/journal.pone.0142409 (PMC4642958; doi:10.1371/journal.pone.0142409)

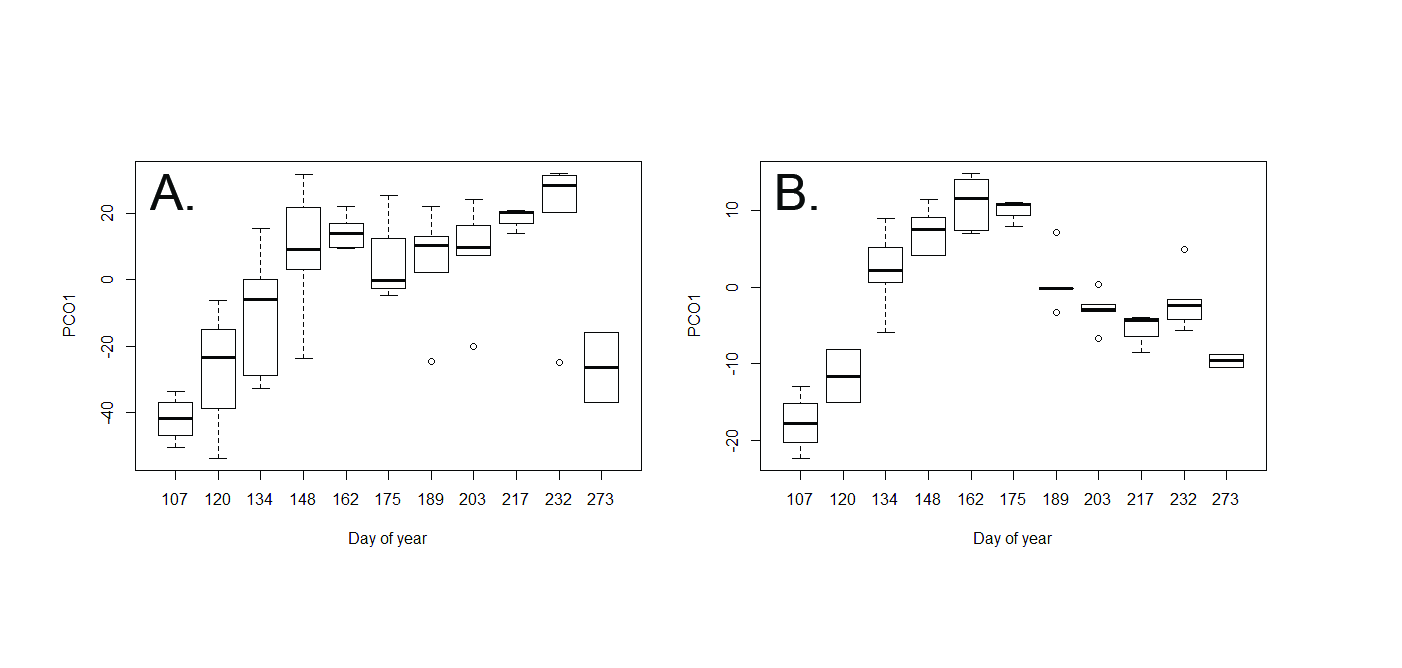

Supplement: S1 Fig — Plot depicts primary axis of principal coordinate analysis (PCoA) for relative abundance data. In PCoA, the first axis accounts for the greatest amount of variation in the dataset. The effect of time in each dataset was tested using PERMANOVA (P < 0.001 in both cases). (TIF) [file pone.0142409.s001.tif]

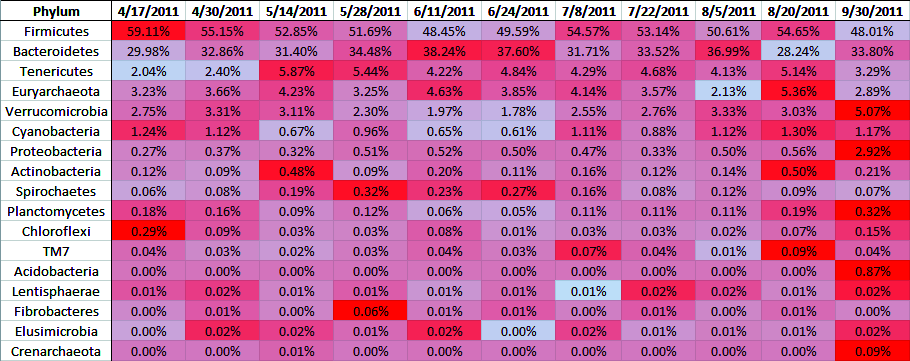

Supplement: S3 Table — Numbers indicate mean relative abundance. Colors indicate z-score, with red representing positive z-scores, blue representing negative z-scores, and brightness of color representing absolute value of z-scores. (TIF) [file pone.0142409.s004.tif]
